# Delayed Mechanical Response to Chemical Kinetics in Self-Oscillating Hydrogels Driven by the Belousov–Zhabotinsky Reaction

**DOI:** 10.1021/acs.macromol.1c00402

**Published:** 2021-07-02

**Authors:** Tunde Geher-Herczegh, Zuowei Wang, Tsukuru Masuda, Ryo Yoshida, Nandini Vasudevan, Yoshikatsu Hayashi

**Affiliations:** † Biomedical Sciences and Biomedical Engineering, School of Biological Sciences, University of Reading, Reading RG6 6DH, U.K.; ‡ Department of Mathematics and Statistics, University of Reading, Reading RG6 6AX, U.K.; § Department of Bioengineering, School of Engineering, The University of Tokyo, Bunkyo-ku 113-8656, Japan; ∥ Department of Materials Engineering, School of Engineering, The University of Tokyo, Bunkyo-ku 113-8656, Japan

## Abstract

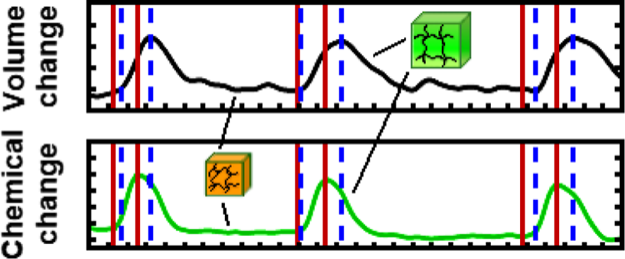

We show experimentally that chemical and mechanical self-oscillations in Belousov–Zhabotinsky hydrogels are inherently asynchronous, that is, there is a detectable delay in swelling–deswelling response after a change in the chemical redox state. This phenomenon is observable in many previous experimental studies and potentially has far-reaching implications for the functionality and response time of the material in future applications; however, so far, it has not been quantified or reported systematically. Here, we provide a comprehensive qualitative and quantitative description of the chemical-to-mechanical delay, and we propose to explain it as a consequence of the slow nonequilibrium swelling–deswelling dynamics of the polymer material. Specifically, standard hydrogel pieces are large enough that transport processes, for example, counterion migration and water diffusion, cannot occur instantaneously throughout the entire gel piece, as opposed to previous theoretical considerations. As a result, the volume response of the polymer to a chemical change may be governed by a characteristic response time, which leads to the emergence of delay in mechanical oscillation. This is supported by our theoretical calculations.

## Introduction

Smart polymer materials that are nonliving yet exhibit complex “life-like” or biomimetic behaviors have been the focus of intensive research over the past decades, in the quest to broaden our understanding of how living systems function and how life could have emerged.^1−4^ One branch of such smart materials is the extensively studied Belousov–Zhabotinsky (BZ) self-oscillating hydrogels, first synthesized in the 1990’s by Yoshida et al.,^5^ that are capable of exhibiting a rich variety of physical–chemical and biomimetic behaviors^6−9^ and show great promise as potential soft actuators, drug delivery systems, and other applications.^10,11^ They demonstrate spontaneous periodic swelling–deswelling changes known as chemomechanical self-oscillation, reminiscent of the rhythmic beating of cardiac cells, by utilizing the well-known BZ chemical reaction. A key reactant of this reaction, a metal catalyst (in this case, a ruthenium tris bipiridyne complex), is covalently bound to the polymer chain as a pendant group, while participating in the redox oscillation (changing between Ru^2+/3+^ states). As a result, all periodic redox changes of these groups lead to rising and falling in polymer charge density, which in turn induces excess counterion migration and osmotic pressure changes and prompts water to enter or leave the polymer network, making it swell or deswell.

Depending on the size, composition, and other parameters of BZ hydrogel systems, a rich variety of temporal and spatial behaviors may be observed. Chemical wave propagation, a phenomenon in excitable nonlinear systems such as the BZ reaction, readily emerges in self-oscillating hydrogels as well, provided that the size of the gel is large enough (several millimeters).^12^ By controlling the exact shape and size of gel samples, various two-dimensional patterns have been shown to evolve over time.^13^ On the other hand, when samples are cut to small pieces (typically sub-millimeter, smaller than the wavelength of the propagating chemomechanical wave), another type of behavior, isotropic volume oscillation, becomes possible, that is, the gel swells and deswells homogeneously in all spatial directions.^14^

Although BZ hydrogels have been studied for over two decades now, there are still critical questions regarding their underlying physical–chemical mechanism and behavior that remain open until today, yet should be explored in order for potential future applications such as soft actuators to be realized. The mechanism of self-oscillation has been discussed to some extent in the framework of a chemical oscillation coupled to a mechanical response, that is, C–M coupling (see e.g., study by Sasaki et al.^15^). Coupled oscillations are abundant in natural systems, when two or more periodic processes interact and influence each other, and the roles of leading and following oscillations may not always be straightforward.^16^ Since BZ gels are fundamentally stimuli-responsive materials, in such systems, it is trivial that changes in the chemical environment are required in order for the hydrogel to undergo volume changes; therefore, C–M coupling here is primarily driven by the chemical reaction. Stimuli-responsive hydrogels of other types and compositions have also been successfully synthesized and shown to produce similar swelling–deswelling self-oscillation in chemical reactions other than the BZ such as pH-oscillators, supporting this assessment regarding leading and following roles.^17,18^ However, experimental evidence has also been found for chemomechanical self-oscillation in a nonoscillatory chemical reaction, where interestingly the dynamics emerge from the enhanced size-change mechanical feedback of the hydrogel.^19^

We propose that there is a yet largely unexplored but certainly prevalent and significant basic feature of chemomechanical self-oscillation in BZ gels that not only shines new light on its fundamental physical–chemical mechanism but also could have potentially far-reaching consequences for future applications of the material. An extensive review, conducted by our group, of published experimental graphs showing the corresponding chemical and mechanical oscillations of BZ gels, reveals a consistent small degree of asynchronicity, specifically, volume change appears to be delayed with respect to chemical shifts (see Table 1 for examples where this was observed). To paraphrase, the mechanical response of the hydrogel to chemical stimuli is clearly not instantaneous but only follows after a detectable lag. To the best of our knowledge, this chemical–mechanical delay has only been mentioned once before,^15^ but no detailed quantitative characterization or compelling theoretical explanation was offered for the phenomenon. However, should BZ hydrogels be considered for advanced soft actuators or other applications, such a delay would be a critical factor in the device’s response time. Therefore, in this study, we intend to fully quantify the emerging chemical–mechanical delay experimentally in modern self-oscillating hydrogels and hypothesize that it conclusively results from the slow nonequilibrium swelling–deswelling dynamics of the polymer network, for which we provide supporting theoretical results from modeling calculations.

**Table 1:** Examples from Previous Studies Where a Delay is Observable between Chemical Change and Mechanical Response in Self-Oscillating Hydrogels

Publication	Figure
Yoshida et al. (1995)^20^	Figure 3
Yoshida et al. (2000)^14^	Figures 2, 4, and 5
Yoshida et al. (2003)^21^	Figure 3
Sasaki et al. (2003)^15^	Figures 1-3
Zhang et al. (2012)^22^	Figure 3

## Experiments

### Materials and Hydrogel Preparation

**Figure 1:**
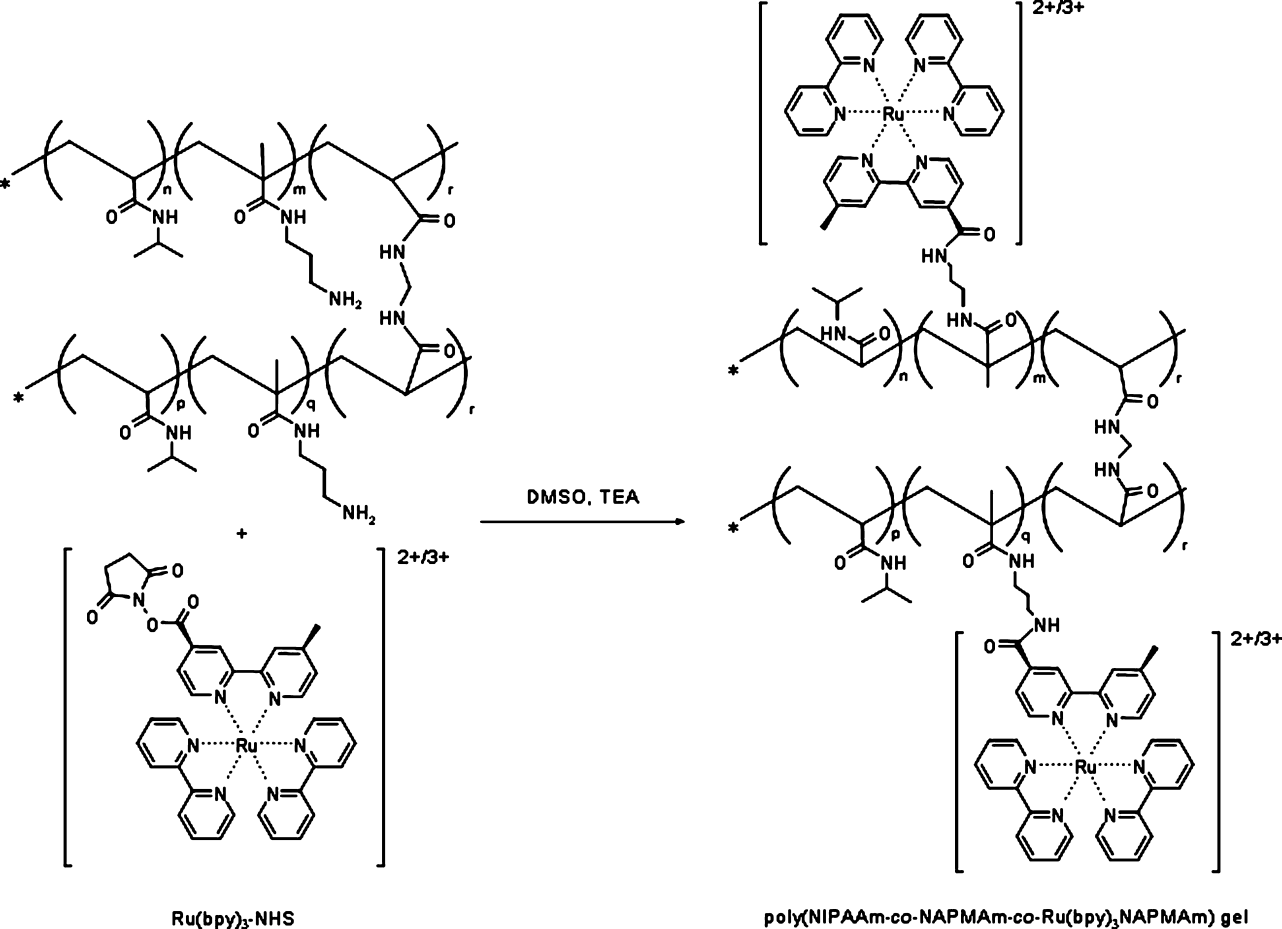
Chemical structure of poly(NIPAAm-*co*-NAPMAm-*co*-Ru(bpy)_3_NAPMAm) BZ hydrogels, synthesized following the procedure by Masuda et al.^23^ Hydrogel preparation is performed in two stages. First, a standard MBAAm cross-linked poly(NIPAAm-*co*-NAPMAm) hydrogel is synthesized by radical copolymerization (top left in the figure), which is then gradually transferred into DMSO. Gel pieces are then immersed in Ru(bpy)_3_-NHS solution in DMSO; triethylamine may be added to aid the reaction, although it is not required. The succinimidyl ester group of Ru(bpy)_3_-NHS reacts with the primary amine pendant groups of the copolymer backbone; thus, the catalyst covalently binds to the polymer network (full structure on the right hand side of the figure).

Self-oscillating hydrogel samples were prepared following the procedure developed by Masuda et al.^23^ We note that this is a relatively more recent two-stage synthesis method than the original BZ gel used by most studies and has a number of advantages in terms of using less harsh polymerization conditions and providing better control over the catalyst concentration in the polymer network. In the first stage, a NIPAAm (*N*-isopropylacrylamide)-based copolymer hydrogel is prepared with added primary amine pendant groups, provided by NAPMAm (*N*-3-(aminopropyl)methacrylamide) monomers; these pendant groups then serve as reactive sites in the second stage for the introduction of the metal redox catalyst, in this case, the bis(2,2′-bipyridine) (1-(4′-methyl-2,2′-bipyridine-4-carbonyloxy)-2,5-pyrrolidinedione)ruthenium(II) bis(hexafluorophosphate) complex or Ru(bpy)_3_-NHS for short (see Figure 1). Choosing the NIPAAm/NAPMAm ratio enables the tuning of metal catalyst concentration in hydrogel samples since the ruthenium complex succinimidyl ester can only bind to the NAPMAm amine groups.

For our experiments, 0.5–1.0 mm sheets of poly(NIPAAm-*co*-NAPMAm) gels were prepared first by radical copolymerization, using NIPAAm (Sigma-Aldrich, purified by recrystallization from toluene/hexane before use) and NAPMAm (Polysciences Europe, used without purification), with 5, 10, and 20 mol % NAPMAm/NIPAAm monomer ratios; the *N*,*N*′-methylenebisacrylamide cross-linker and TEMED and APS reagents were also purchased from Sigma-Aldrich. The basic hydrogel was then washed with water for 2 days; then, the solvent was gradually exchanged to dimethyl sulfoxide (DMSO) (Fisher) over the course of several days. In order to introduce the ruthenium(II) complex groups into the polymer network, hydrogel sheets were cut up into smaller pieces and then immersed in a 70 mM solution of Ru(bpy)_3_-NHS (Trylead Chemical Technology Co Ltd., China) in DMSO and left to react for 24 h. The aim of applying Ru(bpy)_3_-NHS in excess is to ensure that all NAPMAm amine groups of the polymer become saturated with the metal catalyst, thus obtaining 5, 10, and 20% relative catalyst concentrations. Next, the gel pieces were washed with DMSO for a day; then, the solvent was gradually exchanged back to water, after which the BZ gels were ready for experiments.

### Methods

Experiments were performed at 20 ± 0.2 °C in a Clifton unstirred water bath, freshly after gel preparation was finished. Swelling–deswelling self-oscillation was recorded using a Dino-Lite AM4113ZT USB microscope, connected to a Raspberry Pi computer and operated by custom-written Python codes, taking a time-lapse image series with a 10 s sampling rate. In order to initiate oscillation, hydrogel pieces, cut to 0.5–1.0 mm size, were immersed in a catalyst-free BZ mixture of 0.700 M HNO_3_, 0.084 M malonic acid, and 0.104 M NaBrO_3_, freshly prepared for each experiment. Such small gel sizes were chosen in order to elicit consistent isotropic swelling–deswelling behavior across all hydrogel compositions. When samples were placed in the outer solution, time lapse recording was initiated and continued for several hours.

### Data Processing

**Figure 2:**
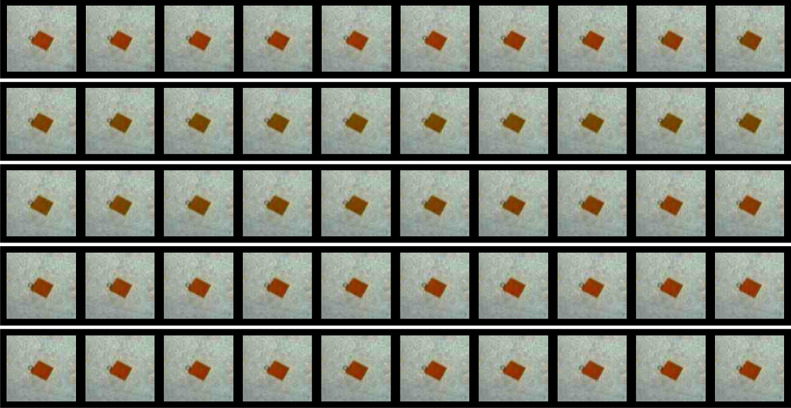
Color change during chemomechanical oscillation in a BZ self-oscillating hydrogel piece (5% catalyst concentration sample). Images have been cropped and displayed to show one oxidation/swelling peak taking around 9 min. Note that the size change is relatively small and hence not directly observable visually in these images; however, it is revealed clearly after analysis. These images correspond to the data presented in Figure 3 graphs 1A,B and Figure 5, specifically the color/size peaks appearing around 4000 s.

Image processing and data analysis were performed using custom-written scripts in Wolfram Mathematica, using a number of practical in-built tools. In order to reduce the overall processing time, all images in the time series were first cropped to show a suitable small section with the gel piece in front of a gray background. This cutout was analyzed at each time point, pixel by pixel, to distinguish between pixels belonging to the gel and the background, based on their RGB values. Due to their different color levels, especially concerning the blue channel, a threshold could be set to separate gel and background pixels efficiently and reliably. Further conditions were also applied for the red and green values to make the separation even more precise. Once all gel pixels were extracted for each time point, their green values were averaged and plotted to reveal chemical oscillations. In small enough gel pieces such as our samples where isotropic chemomechanical oscillation occurred, the gel color would be typically fairly uniform across the entire observed surface at a time and therefore could be safely averaged. Due to the typical red and green colors of the reduced and oxidized forms of the ruthenium complex, respectively, a substantial spike in the average green value of the gel was interpreted as oxidation of the sample (see Figure 2 for an example cropped image series displaying a typical color change, corresponding to one oxidation peak).

Similarly, the size change would happen uniformly during isotropic swelling–deswelling, and therefore, analyzing the apparent 2D surface of gel samples could reveal mechanical oscillations. After color thresholding, the number of gel pixels was counted at each time point to obtain a size value and recalculated to mm^2^ after calibration. Finally, in order to reduce noise, a Gaussian filter was applied to both chemical and mechanical oscillation data sets for more precise results.

## Results and Discussion

### Catalyst Concentration-Dependent Gel Behavior

**Figure 3:**
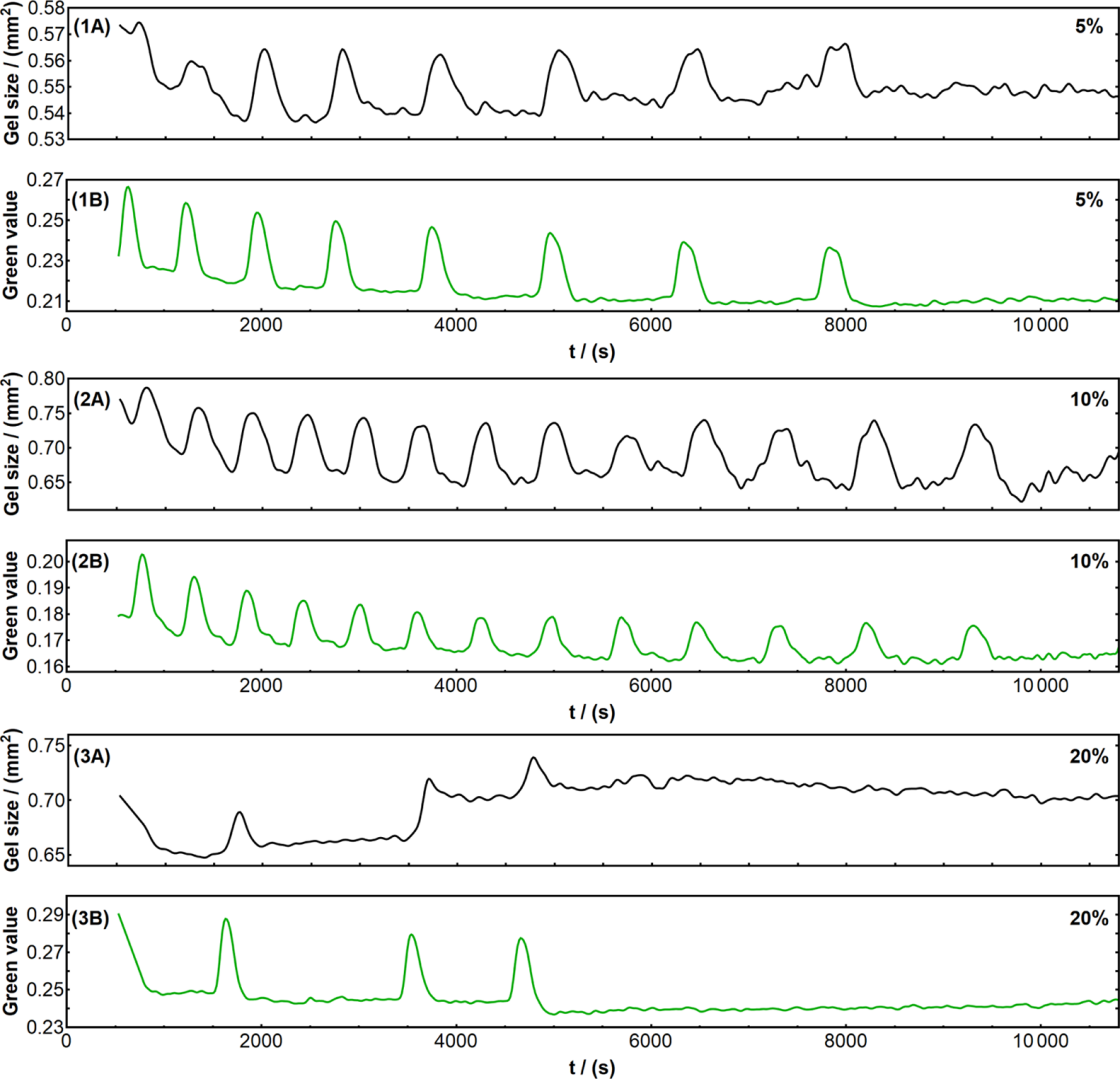
Time series curves showing examples of chemical and mechanical oscillation in various self-oscillating gel samples. Black curves show mechanical oscillation and correspond to the gel size change over time, extracted from time lapse image series as the number of pixels belonging to the gel sample and then recalculated to mm^2^ after image calibration. Green curves show chemical oscillation, revealed by the color change in the gel sample over time and extracted as the green value of the gel piece (obtained as 0–255 values from images and then normalized to fall between 0 and 1). Graphs 1A,B: mechanical and chemical oscillation, respectively, in a gel sample of 5% catalyst concentration. Graphs 2A,B: mechanical and chemical oscillation, respectively, in a gel sample of 10% catalyst concentration. Graphs 3A,B: mechanical and chemical oscillation, respectively, in a gel sample of 20% catalyst concentration.

For all three compositions of BZ hydrogels synthesized (5, 10, and 20% catalyst concentrations), seven to eight samples were analyzed quantitatively and averaged to investigate their oscillatory behaviors. Figure 3 shows example sets of chemical and mechanical time series for each concentration.

**Figure 4:**
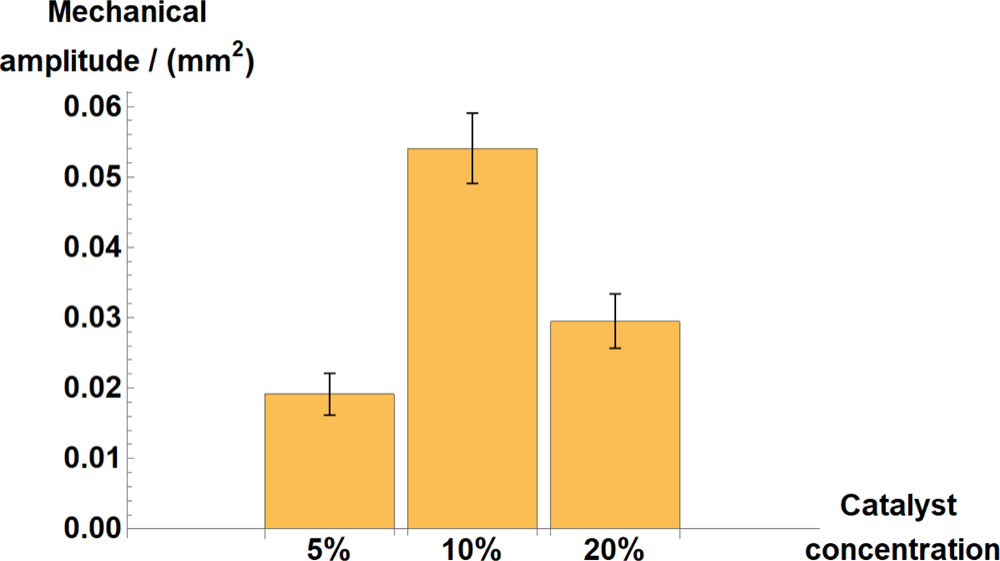
Mechanical or swelling–deswelling amplitude of various BZ hydrogels with different ruthenium contents. The catalyst concentration is expressed in %, referring to the relative molar ratio of the monomer to which the complex is bound (see the synthesis method for full details). Average results are presented as the mean and standard deviation from 7 to 8 samples for each catalyst concentration.

Regular oscillation tended to expire in a few hours after initiation (i.e., the moment when gel pieces were immersed in the catalyst-free BZ solution); therefore, for statistical comparison, all emerging oscillation peaks in the first 3 h (10800 s) were analyzed for each sample (time series); furthermore, since several minutes were required at the beginning of each experiment to arrange the gel samples in the solution under the camera, the first 15 min (900 s) were also excluded from analysis for consistency. Chemical oxidation peaks and the corresponding isotropic swelling–deswelling were routinely observed in all compositions; however, differences in parameters such as the oscillation period, amplitude, and duration emerged due to the different amounts of ruthenium complex in the polymer mesh. Samples of 5 and 10% catalyst concentrations displayed similar behavior as reported originally by Masuda et al.^23^ concerning the oscillation period, which kept increasing over time. In addition, 10% samples were found to have shorter periods, in other words, faster oscillation, than 5% samples, which is in good agreement with previous self-oscillating hydrogel studies.^14^ Higher catalyst concentration is also known to cause a larger swelling–deswelling amplitude,^14,22^ defined here as the difference between the bottom and the top of a swelling peak, which was again exhibited by our data in the 5–10% gels, see Figure 4.

Interestingly, gel samples with 20% catalyst concentration did not follow these established trends and yielded substantially disrupted oscillations (see Figure 3 graphs 3A,B, e.g.), with only one or two isotropic peaks in some samples and no steady oscillation periods. As illustrated by Figure 4 as well, the mechanical amplitude of swelling peaks was significantly smaller, on average, in 20% samples than in 10% ones, which may be explained due to the overcrowded state of the polymer network. In the 20% gel samples, that is, in the case of 4:1 = NIPAAm/NAPMAm molar ratio, each network strand between two adjacent MBAAm cross-linkers is, on average, composed of 29 NIPAAm and 7.25 NAPMAm monomers. Understanding that the ruthenium complex only binds to NAPMAm, this would result in an extremely large number of catalyst complexes for each network strand, even though it might be suspected that not all NAPMAm groups may become saturated in such a crowded mesh.

The average end-to-end distance *R*_e_ and radius of gyration *R*_g_ of a gel network strand can be approximated by those of a free chain with the same chemical composition in the same solvent. Since the NIPAAm and NAPMAm monomers have roughly the same backbone length of *l* ≈ 0.25 nm, we can estimate the strand size in a gel composed of these monomers using the Kuhn length *l*_k_ found in linear poly(isopropylacrylamide) (PNIPAM) in aqueous solution. Recently, Lopez et al. reported an estimated Kuhn length *l*_k_ ≈ 4 ± 1 nm for linear PNIPAM by fitting static and dynamic light scattering (SLS/DLS) data on the chain hydrodynamic radius and *R*_g_ to the ideal worm-like chain model, while previous single-molecule force spectroscopy (SMFS) experiments rendered a much smaller average value of *l*_k_ ≈ 0.7 nm, see ref (24) and references therein. Taking the SLS/DLS value of *l*_k_ ≈ 4 nm, there are *l*_k_/*l* ≈ 16 NIPAAm monomers per Kuhn segment in a linear PNIPAM. In the 20% gel samples we used, the average number of 36.25 NIPAAm/NAPMAm monomers per strand corresponds to *N*_k_ ≈ 2.26 Kuhn segments. The average network strand sizes in the absence of Ru(bpy)_3_ groups can then be estimated as *R*_e_ ≈ *N*_k_^1/2^*l*_k_ ≈ 6.02 nm and *R*_g_ ≈ 2.46 nm using the Gaussian chain model^25^ and *R*_e_ ≈ 5.32 nm and *R*_g_ ≈ 1.85 nm using the ideal worm-like chain model.^24,26^ If the SMFS value of *l*_k_ ≈ 0.7 nm is used instead, each strand would have *N*_k_ ≈ 12.95 Kuhn segments, giving estimated strand sizes *R*_e_ ≈ 2.52 nm and *R*_g_ ≈ 1.03 nm using the Gaussian chain model and *R*_e_ ≈ 2.47 nm and *R*_g_ ≈ 0.97 nm using the ideal worm-like chain model. On the other hand, the Ru(bpy)_3_ complex takes a spherical shape with an estimated diameter of approximately 1.3 nm, which is of comparable order to the gel strand size. When there are multiple Ru(bpy)_3_ groups attached to the 7.25 NAPMAm monomers on each strand, the excluded volume interactions between these groups will strongly suppress the fluctuations of the polymer strands and consequently that of the entire gel. Therefore, the mechanical oscillation amplitude of the BZ gels cannot grow monotonically with the increase in catalyst concentration, even though the electrostatic repulsion and osmotic effects continuously increase. This can partly explain the decrease in the mechanical amplitude when the catalyst concentration is increased from 10 to 20%, as shown in Figure 4.

We also note that the binding of the bulky Ru(bpy)_3_ groups to the NAPMAm monomers might increase the monomer frictional coefficients and so slightly slow down the relaxation dynamics of the polymer backbones, which, combined with significant effects of the friction of the solvent with the polymer mesh, could lead to a diminished volume response of the gel to changes in osmotic pressure. It is the complicated interplay among the excluded volume, effective monomer friction, polymer elasticity, solvent friction, electrostatic repulsion, and osmotic pressure which results in a decreased mechanical oscillation amplitude at high catalyst concentrations.

### Chemomechanical Delay

**Figure 5:**
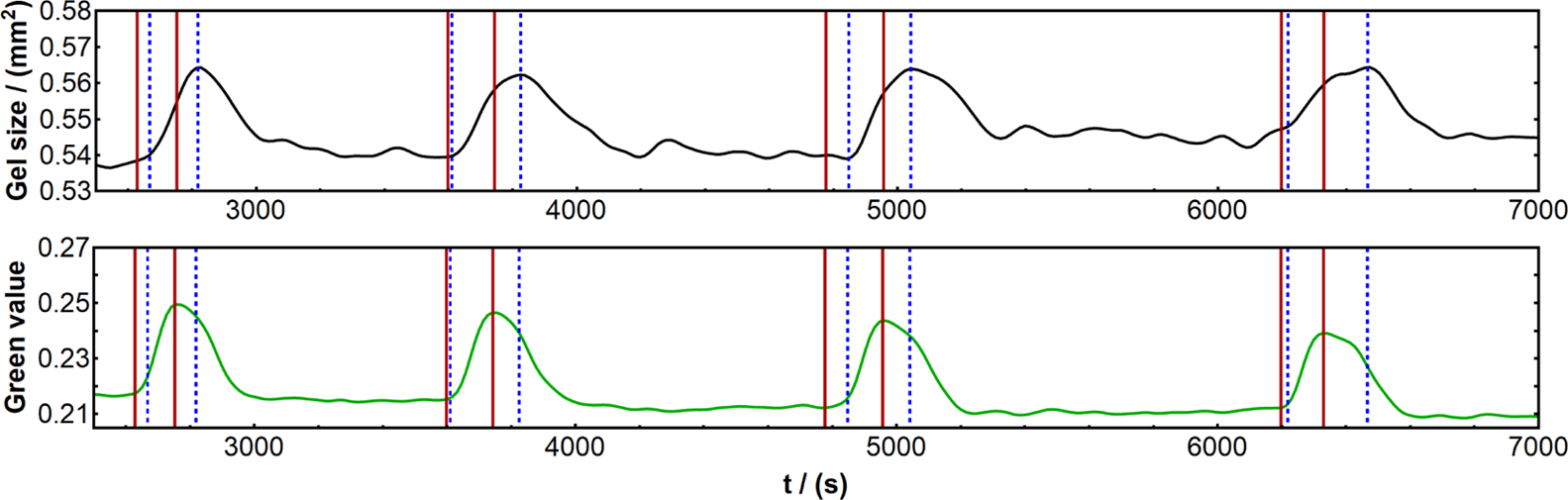
Emergence of chemomechanical CM delay (i.e., delayed mechanical response to the chemical redox change), illustrated by a 5% catalyst concentration gel sample. Top black curve: mechanical oscillation (gel size change over time). Bottom green curve: chemical oscillation (change in green value of the gel color over time, obtained as 0–255 values from images and then normalized to fall between 0 and 1). Vertical lines have been drawn at the starting points of oxidation/swelling (=feet of the peaks) and reduction/deswelling (=top of the peaks) to show the existence of the delay; red lines correspond to the chemical oscillation, and blue dashed lines correspond to the mechanical oscillation.

**Figure 6:**
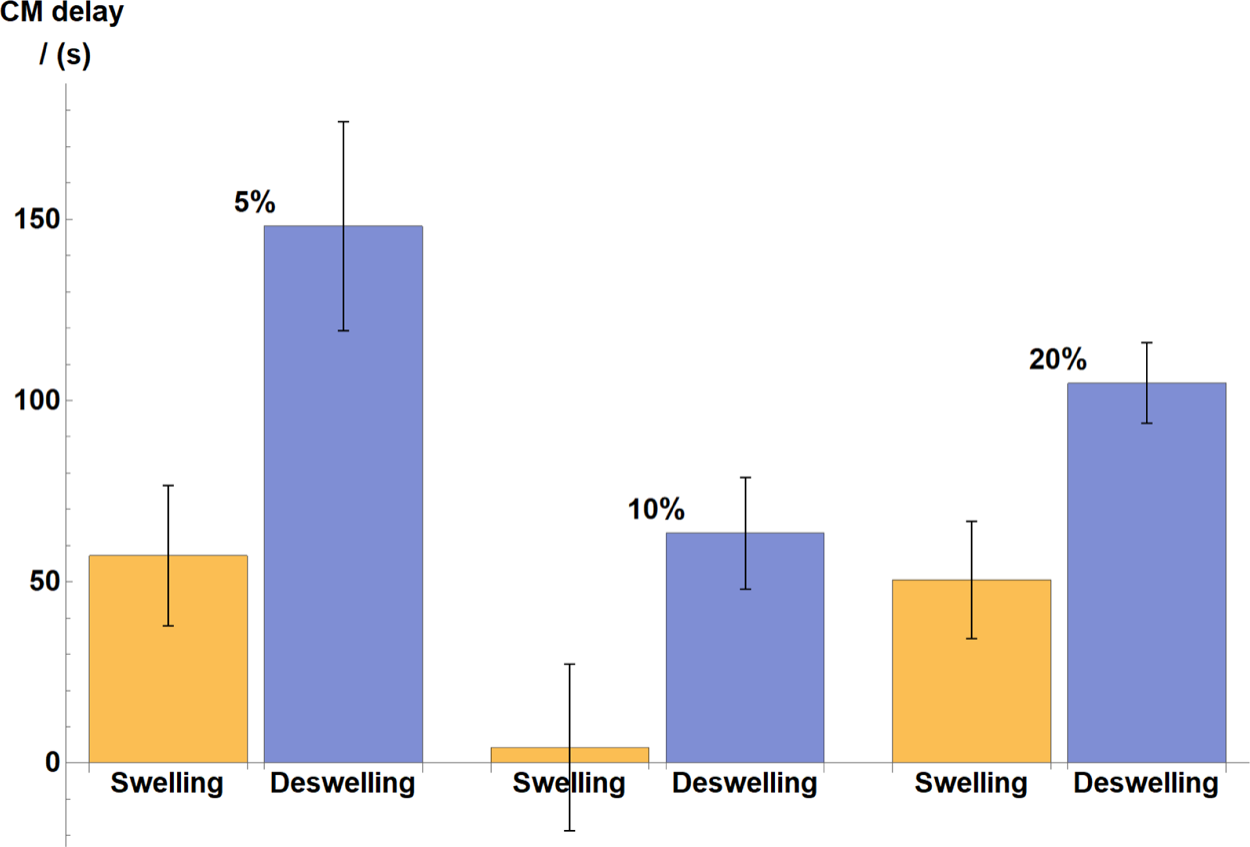
Statistical summary of the chemomechanical CM delay results, in BZ gels with 5, 10, and 20% catalyst concentrations. The catalyst concentration is expressed in %, referring to the relative molar ratio of the monomer to which the complex is bound (see the synthesis method for full details). Average results are presented as the mean and standard deviation from seven to eight samples for each catalyst concentration. Comparison is drawn between swelling and deswelling delays for each concentration, that is, the time required for swelling to start in response to oxidation and deswelling to start in response to reduction, respectively.

Next, time series for all three catalyst concentrations were analyzed to investigate the synchronicity of chemical and mechanical oscillations. In all samples, the volume (apparent size) change was observed to be consistently delayed relative to the chemical change, as expected based on our literature review and preliminary experiments (see the illustration in Figure 5). Full quantitative summary of all results can be found in Figure 6, which reveals interesting trends and observations. A significant difference was detected in the swelling and deswelling delays, that is, the hydrogel’s mechanical response to oxidation and reduction, respectively. Deswelling delays were found to be at least twice as long, on average, as swelling delays across all 5–20% catalyst concentrations, indicating that the gels were much slower to respond to reduction than oxidation. This seems to intuitively correspond to fundamental BZ dynamics since reduction itself is a slower process in the reaction system than fast autocatalytic oxidation.

Furthermore, comparing the 5 and 10% ruthenium concentration samples, it is clear that the higher catalyst content results in shorter average delays for both swelling and deswelling. At first, this might be suspected to be a nonspecific consequence of reaction kinetics, that is, the fact that 10% samples yield faster oscillations in general (refer back to Figure 3); however, our analysis shows that the effect is much more substantial than that. Increasing the catalyst concentration from 5 to 10% gives a less than 20% decrease only in the average oscillation period, yet both swelling and deswelling delays fall by around 60–70%. We speculate that this is due to the increased electrostatic interactions causing more intense counterion migration between the hydrogel and the outer solution: in the 10% composition, larger numbers of the Ru(bpy)_3_^2+/3+^ pendant group result in higher charge density on the polymer network (compared to the 5% samples), which then generates a larger difference in the chemical potential of counterions between the outer solution and the gel, as it undergoes redox changes. This in turn affects the chemical potential of water molecules as well, prompting a larger flux of water between the hydrogel and solution phases (i.e., it intensifies the hydration process). The magnitude of volume change is primarily determined by this water flux and the amount of time the system has for water to flow in and out. Therefore, it follows that as the water flux increases with the catalyst concentration, shorter lengths of time will be required for the same amount of volume change to occur; hence, the chemomechanical (CM) delays will be shorter as well.

We note the 20% gel samples again fail to follow the abovementioned trend and both average swelling and deswelling delays are reasonably longer than in 10% samples. This is again believed to be a result of the same excluded volume interactions and overcrowded polymer mesh as explained before with relation to the decrease in mechanical oscillation amplitude.

We propose that CM delays may arise from the interplay among the diffusion rate-limited gel-solvent mixing process, chemical reaction oscillation, and gel volume fraction change, provided that we assume a nonequilibrium nature in the swelling–deswelling response, as opposed to the already existing theoretical considerations in the literature.^27−29^ Previously, BZ gels have been described to have different equilibrium swelling ratios in their fully reduced and oxidized states (see, for instance, the systematic study by Masuda et al. for the newer type of poly(NIPAAm-*co*-NAPMAm-*co*-Ru(bpy)_3_NAPMAm) BZ gels^23^), which results in volume changes when the chemical environment shifts between these redox states. We assert that although this is an adequate macroscopic explanation for the phenomenon, it does not capture the full picture of the underlying physical–chemical processes. Even though these particular BZ gel systems in question tend to have relatively long oscillation periods (on the scale of 10–20 min, depending on reactant concentrations and temperature), the length of time required for ∼1 mm^3^ gel samples to become fully reduced or oxidized would be comparable to these periods or even longer.

Therefore, we postulate that the hydrogel may not reach its equilibrium volumes during chemomechanical oscillation, and our physical–chemical interpretation of CM delays is based on this hypothesis. Although reactants and products diffuse freely between the gel and the outer solution, the chemical reaction is strictly confined to the polymer network due to the immobilized ruthenium complex groups. The volume of hydrogel samples is relatively very small compared to the large reservoir of outer solution (typically around or less than 1 mm^3^ gel pieces in 100 mL of solution), however, still large enough that diffusion within the gel and between the two phases is not instantaneous. When changes in the chemical environment prompt increases or decreases in the osmotic pressure, the system is being driven toward equilibrium volume every moment; however, it cannot reach it before the redox state changes again and shifts the equilibrium. Since chemical oscillation is fundamentally a nonequilibrium phenomenon, a volume change will only be able to chase after it. The appearance of detectable delays follows directly from this: for instance, close to the top of oxidation peaks, the ionic strength is high due to the Ru(bpy)_3_^3+^ groups, which prompts water to enter the polymer mesh; when reduction takes over, ionic strength remains still comparatively high for a short while that drives more water in. Only after a significant proportion of the catalyst groups has become reduced and the ionic strength has fallen below a threshold will the hydrogel network become hydrophobic enough to repel some of its water content and cause it to deswell; hence, a time difference between chemical and mechanical peaks will appear.

### Theoretical Description

Here, we present a phenomenological theoretical model to describe the C–M delay behavior of BZ gel samples that are of sufficiently small sizes and so undergo homogeneous (isotropic) swelling–deswelling oscillations. Previous experiments have observed uniform oscillations in various responsive BZ gel samples with lateral sizes ranging from 0.5 mm to around 1 mm,^13,14,23^ but generating traveling waves in relatively larger samples.^29,30^ The sample sizes used in our experiments fall well into the isotropic oscillation regime.

Our model is adapted from the theoretical framework developed by Yashin et al. for describing the self-oscillation behavior of BZ gels^27−29^ by introducing a delayed response mechanism of the gel volume change in the chemical reaction, to replace the original assumption of instantaneous response. In the theoretical work of Yashin et al., the Oregonator model originally developed for describing BZ reactions in simple solutions^31^ was modified to take into account the effect of gel swelling/deswelling on the reaction kinetics. For a BZ gel with the polymer volume fraction ϕ and so the solvent volume fraction 1 – ϕ, the modified Oregonator model in the dimensionless form was mathematically written in terms of two dimensionless variables, *u* and *v*, measuring the concentrations of the reagent in solution (X = [HBrO_2_] in our system) and oxidized catalyst covalently bound to the polymer backbones (Z = [M_ox_] = [Ru(byp)_3_^3+^]), respectively
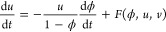
1
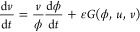
2where the first terms on the right hand side (RHS) of the equations describe the concentration variation due to the swelling/deswelling of the gel. The reaction rate functions *F* and *G* are
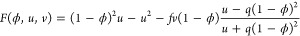
3
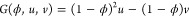
4where *f* is the stoichiometric factor representing the number of bromide ions produced when two oxidized metal ions are reduced. It is treated as a model parameter. The dimensionless parameters *q* and ε are defined in the original Oregonator model using the concentrations of reactants, that is, [BrO_3_^–^], [all oxidizable organic species] and [H^+^], and the five rate constants used in the three original reaction rate equations.^28,31^

At given concentrations *u* and *v*, the equilibrium volume fraction of polymers ϕ is determined by the condition of zero net osmotic pressure Π in the gel
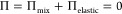
5

The osmotic pressure contribution due to the mixing between the polymer network and solvent is given by^27^
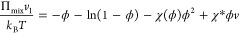
6where *k*_B_ is the Boltzmann constant, *T* is temperature, and *v*_1_ is the volume of a polymer monomer. The first three terms on the RHS of eq 6 are from the standard Flory–Huggings (FH) theory with the FH parameter χ(ϕ) = χ_0_ + χ_1_ϕ where χ_0_ is temperature-dependent and χ_1_ is a constant. The coupling between the BZ reaction and gel dynamics is modeled by the last term on the RHS of eq 6 with χ* > 0 which describes the hydrating effect of the oxidized catalyst on the swelling of the gel.^27^ The contributions of the electrostatic interactions between the catalyst ions and the translational entropy of free counterions to the osmotic pressure are neglected in their model.

In the absence of external forces, the gel samples of sufficiently small sizes can be considered to swell/deswell uniformly in all three dimensions. The Flory model for gel elasticity then gives the elastic osmotic contribution
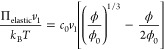
7where *c*_0_ is the number density of elastic strands in the polymer network and ϕ_0_ is the polymer volume fraction in the undeformed state. For sufficiently small BZ gel samples, the transport of the solvent in and out of the gel region is considered to be instantaneous. The equilibration of the osmotic pressure Π is also assumed to take place instantaneously at any moment of time. Equations 1, 2, and 5 can then be solved together to provide the time-dependent values of *u*(*t*), *v*(*t*), and ϕ(*t*) for describing the self-oscillation behavior of the BZ gels.^27−29^

In the abovementioned theoretical framework, the polymer volume fraction ϕ was assumed to change with the chemical reaction instantaneously via the coupling term in the osmotic pressure Π_mixing_. The mixing of the solvent with the gel network is however a rate-limited diffusion process depending on many factors such as the compositions of the polymer and solvent which determine the interactions and frictions between different species and the interfacial features between the gel and surrounding solvents that vary with the gel volume change.^29,32,33^ The characteristic time of the gel-solvent mixing or also called the interdiffusion process has been experimentally shown to interplay with the period of chemical oscillations to affect the amplitude of swelling–deswelling oscillations of cubic BZ gel samples with a side length of about 0.5 mm.^14,29^ This mixing process also contributes to the delayed response of the gel volume change to the chemical reaction, as observed in our experiments. The microscopic picture of the mixing kinetics may be investigated by well-designed experiments or computer simulations at the atomic level. Below, we introduce a simple phenomenological description of such a delayed mechanical response. A more realistic and quantitative model can be developed later based on the understanding of the microscopic mechanisms of the delayed responses.

For a BZ gel with a polymer volume fraction ϕ(*t*_0_) at time *t*_0_, the condition of zero net osmotic pressure in eq 5 predicts a polymer volume fraction ϕ_idl_ at *t*_0_ + δ*t* after a time interval δ*t*. Assuming a delayed mechanical response to the osmotic pressure change, the volume fraction of the gel undergoes a smooth transition from ϕ(*t*_0_) to ϕ_idl_, instead of an abrupt jump. This process can be described mathematically using the logistic model
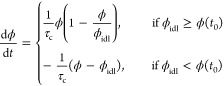
8where τ_c_ is the characteristic response time. The differential eqs 1, 2, and 8 are solved numerically to provide *u*(*t*), *v*(*t*), and ϕ(*t*) in the presence of delayed mechanical response. At time *t* = *t*_0_ + δ*t*, the exact solution to eq 8 is
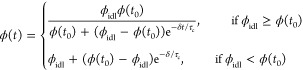
9

If τ_c_ ≫ δ*t*, the response will be significantly delayed, while if τ_c_ ≪ δ*t*, the response is essentially instantaneous, as assumed in previous theoretical models by Yashin et al.^27−29^

**Figure 7:**
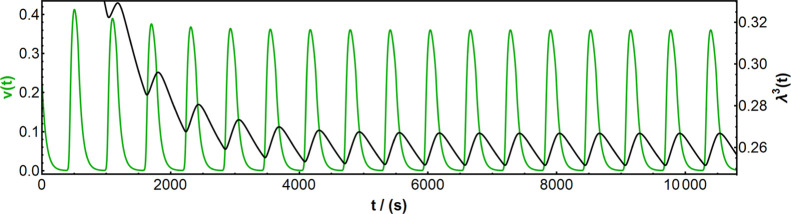
Theoretical calculation results showing the emergence of chemical–mechanical delay. The green curve denotes the time-dependent change in the concentration of *v*(*t*), that is, chemical oscillation, and the black curve denotes the change in dimensionless polymer volume λ^3^(t) = ϕ_0_/ϕ(t), that is, mechanical oscillation. For effective comparison with experimental curves, the first 3 h (10800 s) are plotted. CM delays in both the swelling and deswelling responses are clearly visible in the calculated curves; furthermore, they also capture the observed experimental trends where the swelling delay is much shorter than the deswelling delay.

In this work, the FH parameters and the volume fraction of undeformed BZ gel are taken from the literature as χ_0_ = 0.338, χ_1_ = 0.518, and ϕ_0_ = 0.139.^28^ We note that the exact values of these parameters will rely on the compositions of the gel and solutions and the sample preparation conditions. Varying these values within the experimentally relevant range will only change the model calculation results quantitatively but not qualitatively. The Oregonator model parameters are chosen to be *q* = 1.32 × 10^–4^, ε = 0.0955, and *f* = 0.55, as guided by the gel sample parameters used in our experiments. The coupling parameter is taken to be χ* = 0.02. Numerical solution of the theoretical model using these parameter values produces the self-oscillation behavior in *u*, *v*, and the gel size in reasonably good agreement with the experimental observations. The average self-oscillation period, as measured by the peak-to-peak time difference, is given to be 622 s that is reasonably close to the experimental value. The C–M delay behavior is semiquantitatively captured using a characteristic time τ_c_ = 100 s, as shown in Figure 7. Comparing these modeling results with experimental curves (Figure 3 graphs 1A,B and 2A,B, as well as Figure 5), we are able to observe the same general tendencies: first, the calculated dimensionless polymer volume shows a prominent initial decrease over the first few oscillation cycles, which can be also clearly observed in experimental data sets. Second, there is an asymmetry in the volume oscillation peaks which correspond to shorter swelling delays and significantly longer deswelling delays. The asymmetric kinetics of swelling–deswelling is in good qualitative agreement with previous theoretical and experimental studies concerning poly(*N*-isopropylacrylamide)-type and similar hydrogels, where it was found that the so-called shrinkage barrier effect could slow down deswelling, see, for example, refs (32)–^34^. However, we hypothesize that in our dynamic nonequilibrium system, the kinetics of the BZ reaction have a much more substantial effect on the asymmetry, since swelling is triggered by fast autocatalytic oxidation, whereas reduction leading to deswelling is inherently slower. Furthermore, earlier measurements of swelling–deswelling kinetics in this specific polymeric system by Masuda et al.^23^ did not indicate hindered shrinkage at all in such small gel pieces. All in all, these theoretical results indicate that the delayed mechanical response mechanism to the chemical reactions is needed for understanding and theoretically describing the self-oscillation behavior of the BZ gels.

We note that the theoretical work presented in this and previous work did not take into account the excluded volume and electrostatic repulsion effects between the Ru(bpy)_3_ groups or the translational entropy of the charged reactants and counterions. The usage of the coupling term in eq 6 for describing the hydrating effect and the logistic model in eq 9 for the delayed mechanical response is at the phenomenological level. Microscopic understanding of these dynamic processes and the underlying mechanisms is still needed.

## Conclusions

In this study, we have shown experimentally that the autonomous chemomechanical self-oscillation in BZ hydrogels has an inherently delayed swelling–deswelling mechanical response with regard to periodic redox changes. Owing to the fundamental BZ dynamics, the swelling response to oxidation is always found to be shorter than the deswelling response to reduction. The average length of CM delay also highly depends on the concentration of the catalyst, that is, the covalently bound Ru(bpy)_3_ complex groups. Between 5 and 10% ratios, the higher concentration yields shorter delays; however, samples with 20% catalyst concentration show longer delays and significantly disrupted oscillations, which may be explained by the excluded volume interactions between Ru(bpy)_3_ groups in the overcrowded polymer network.

In order to explain the emergence of CM delays, we provided a nonequilibrium theoretical framework and performed numerical calculations. Despite having small enough hydrogel pieces (<1 mm) that went through isotropic swelling–deswelling, the polymer would not be capable of instantaneous mechanical response, that is, the volume change would be gradual and time-dependent. This model successfully reproduced the appearance of chemomechanical delay, as well as the experimental tendency of shorter swelling and longer deswelling responses. Our theoretical framework not only builds on previously published and validated self-oscillating gel models but also expands them to describe this so far mostly unreported but nonetheless crucial fundamental physical–chemical behavior of BZ gels. Understanding the source and properties of chemomechanical delay is essential for any future applications; therefore, it can aid further research into functional polymers and potential soft actuators.

## References

[ref1] Balazs A. C.; Epstein I. R. Emergent or Just Complex?. Science 2009, 325, 1632–1634. 10.1126/science.1178323. 19779180

[ref2] He X.; Aizenberg M.; Kuksenok O.; Zarzar L. D.; Shastri A.; Balazs A. C.; Aizenberg J. Synthetic homeostatic materials with chemo-mechano-chemical self-regulation. Nature 2012, 487, 214–218. 10.1038/nature11223. 22785318

[ref3] Lim H. L.; Hwang Y.; Kar M.; Varghese S. Smart hydrogels as functional biomimetic systems. Biomater. Sci. 2014, 2, 603–618. 10.1039/c3bm60288e. 32481841

[ref4] Yu C.; Guo H.; Cui K.; Li X.; Ye Y. N.; Kurokawa T.; Gong J. P. Hydrogels as dynamic memory with forgetting ability. Proc. Natl. Acad. Sci. U.S.A. 2020, 117, 18962–18968. 10.1073/pnas.2006842117. 32719128 PMC7431076

[ref5] Yoshida R.; Takahashi T.; Yamaguchi T.; Ichijo H. Self-Oscillating Gel. J. Am. Chem. Soc. 1996, 118, 5134–5135. 10.1021/ja9602511.

[ref6] Yuan P.; Kuksenok O.; Gross D. E.; Balazs A. C.; Moore J. S.; Nuzzo R. G. UV patternable thin film chemistry for shape and functionally versatile self-oscillating gels. Soft Matter 2013, 9, 1231–1243. 10.1039/c2sm27100a.

[ref7] Zhang Y.; Zhou N.; Akella S.; Kuang Y.; Kim D.; Schwartz A.; Bezpalko M.; Foxman B. M.; Fraden S.; Epstein I. R.; Xu B. Active cross-linkers that lead to active gels. Angew. Chem. Int. Ed. 2013, 52, 11494–11498. 10.1002/anie.201304437. 24030921

[ref8] Kim Y. S.; Tamate R.; Akimoto A. M.; Yoshida R. Recent developments in self-oscillating polymeric systems as smart materials: From polymers to bulk hydrogels. Mater. Horiz. 2017, 4, 38–54. 10.1039/c6mh00435k.

[ref9] Aizenberg M.; Okeyoshi K.; Aizenberg J. Inverting the Swelling Trends in Modular Self-Oscillating Gels Crosslinked by Redox-Active Metal Bipyridine Complexes. Adv. Funct. Mater. 2018, 28, 1704205 10.1002/adfm.201704205.

[ref10] Isakova A.; Novakovic K. Oscillatory chemical reactions in the quest for rhythmic motion of smart materials. Eur. Polym. J. 2017, 95, 430–439. 10.1016/j.eurpolymj.2017.08.033.

[ref11] Anna I.; Katarina N. Pulsatile release from a flat self-oscillating chitosan macrogel. J. Mater. Chem. B 2018, 6, 5003–5010. 10.1039/c8tb00781k. 32255072

[ref12] Miyakawa K.; Sakamoto F.; Yoshida R.; Kokufuta E.; Yamaguchi T. Chemical waves in self-oscillating gels. Phys. Rev. E: Stat. Phys., Plasmas, Fluids, Relat. Interdiscip. Top. 2000, 62, 793–798. 10.1103/physreve.62.793. 11088535

[ref13] Chen I. C.; Kuksenok O.; Yashin V. V.; Moslin R. M.; Balazs A. C.; Van Vliet K. J. Shape- and size-dependent patterns in self-oscillating polymer gels. Soft Matter 2011, 7, 3141–3146. 10.1039/c0sm01007c.

[ref14] Yoshida R.; Tanaka M.; Onodera S.; Yamaguchi T.; Kokufuta E. In-phase synchronization of chemical and mechanical oscillations in self-oscillating gels. J. Phys. Chem. A 2000, 104, 7549–7555. 10.1021/jp0011600.

[ref15] Sasaki S.; Koga S.; Yoshida R.; Yamaguchi T. Mechanical Oscillation Coupled with the Belousov–Zhabotinsky Reaction in Gel. Langmuir 2003, 19, 5595–5600. 10.1021/la0270035.

[ref16] Stankovski T.; Pereira T.; McClintock P. V.; Stefanovska A. Coupling functions: Universal insights into dynamical interaction mechanisms. Rev. Mod. Phys. 2017, 89, 045001 10.1103/revmodphys.89.045001. PMC6834002 31656134

[ref17] Labrot V.; De Kepper P.; Boissonade J.; Szalai I.; Gauffre F. Wave patterns driven by chemomechanical instabilities in responsive gels. J. Phys. Chem. B 2005, 109, 21476–21480. 10.1021/jp055095b. 16853785

[ref18] Nwosu C. J.; Hurst G. A.; Novakovic K. Genipin Cross-Linked Chitosan-Polyvinylpyrrolidone Hydrogels: Influence of Composition and Postsynthesis Treatment on pH Responsive Behaviour. Adv. Mater. Sci. Eng. 2015, 2015, 1–10. 10.1155/2015/621289.

[ref19] Horváth J.; Szalai I.; Boissonade J.; De Kepper P. Oscillatory dynamics induced in a responsive gel by a non-oscillatory chemical reaction: Experimental evidence. Soft Matter 2011, 7, 8462–8472. 10.1039/c1sm05226h.

[ref20] Yoshida R.; Ichijo H.; Hakuta T.; Yamaguchi T. Self-oscillating swelling and deswelling of polymer gels. Macromol. Rapid Commun. 1995, 16, 305–310. 10.1002/marc.1995.030160412.

[ref21] Yoshida R.; Takei K.; Yamaguchi T. Self-beating motion of gels and modulation of oscillation rhythm synchronized with organic acid. Macromolecules 2003, 36, 1759–1761. 10.1021/ma0259618.

[ref22] Zhang Y.; Li N.; Delgado J.; Zhou N.; Yoshida R.; Fraden S.; Epstein I. R.; Xu B. Structural modulation of self-oscillating gels: Changing the proximity of the catalyst to the polymer backbone to tailor chemomechanical oscillation. Soft Matter 2012, 8, 7056–7061. 10.1039/c2sm25797a.

[ref23] Masuda T.; Terasaki A.; Akimoto A. M.; Nagase K.; Okano T.; Yoshida R. Control of swelling-deswelling behavior of a self-oscillating gel by designing the chemical structure. RSC Adv. 2015, 5, 5781–5787. 10.1039/c4ra10675j.

[ref24] Lopez C. G.; Scotti A.; Brugnoni M.; Richtering W. The Swelling of Poly(Isopropylacrylamide) Near the θ Temperature: A Comparison between Linear and Cross-Linked Chains. Macromol. Chem. Phys. 2020, 220, 1800421 10.1002/macp.201800421.

[ref25] Rubinstein M.; Colby R. H. Polymer Physics, 1st ed.; Oxford University Press: Oxford New York, 2003.

[ref26] Benoit H.; Doty P. Light scattering from non-Gaussian chains. J. Phys. Chem. 1953, 57, 958–963. 10.1021/j150510a025.

[ref27] Yashin V. V.; Balazs A. C. Modeling Polymer Gels Exhibiting Self-Oscillations Due to the Belousov–Zhabotinsky Reaction. Macromolecules 2006, 39, 2024–2026. 10.1021/ma052622g.

[ref28] Yashin V. V.; Balazs A. C. Theoretical and computational modeling of self-oscillating polymer gels. J. Chem. Phys. 2007, 126, 124707 10.1063/1.2672951. 17411152

[ref29] Yashin V. V.; Kuksenok O.; Dayal P.; Balazs A. C. Mechano-chemical oscillations and waves in reactive gels. Rep. Prog. Phys. 2012, 75, 066601 10.1088/0034-4885/75/6/066601. 22790650

[ref30] Yoshida R. Self-oscillating gels driven by the Belousov-Zhabotinsky reaction as novel smart materials. Adv. Mater. 2010, 22, 3463–3483. 10.1002/adma.200904075. 20503208

[ref31] Tyson J. J.; Fife P. C. Target patterns in a realistic model of the Belousov-Zhabotinskii reaction. J. Chem. Phys. 1980, 73, 2224–2237. 10.1063/1.440418.

[ref32] Tanaka T.; Fillmore D. J. Kinetics of swelling of gels. J. Chem. Phys. 1979, 70, 1214–1218. 10.1063/1.437602.

[ref33] Li Y.; Tanaka T. Kinetics of swelling and shrinking of gels. J. Chem. Phys. 1990, 92, 1365–1371. 10.1063/1.458148.

[ref34] Richter A.; Howitz S.; Kuckling D.; Arndt K.-F. Influence of volume phase transition phenomena on the behavior of hydrogel-based valves. Sens. Actuators, B 2004, 99, 451–458. 10.1016/j.snb.2003.12.014.

